# *β*-Elemene Inhibits Cell Proliferation by Regulating the Expression and Activity of Topoisomerases I and II*α* in Human Hepatocarcinoma HepG-2 Cells

**DOI:** 10.1155/2015/153987

**Published:** 2015-06-29

**Authors:** Min Gong, Ying Liu, Jian Zhang, Ya-jie Gao, Ping-ping Zhai, Xi Su, Xiang Li, Yan Li, Li Hou, Xiao-nan Cui

**Affiliations:** ^1^Department of Oncology, The First Affiliated Hospital of Dalian Medical University, Dalian, Liaoning 116011, China; ^2^Department of General Surgery, The First Affiliated Hospital of Dalian Medical University, Dalian, Liaoning 116011, China; ^3^Department of Pathology, School of Basic Medicine, Dalian Medical University, Dalian, Liaoning 116023, China

## Abstract

*Objective*. To investigate the effects of *β*-Elemene (*β*-ELE) on the proliferation, apoptosis, and topoisomerase I (TOPO I) and topoisomerase II*α* (TOPO II*α*) expression and activity of human hepatocarcinoma HepG-2 cells. *Methods*. After treatment with *β*-ELE, morphological alterations of HepG-2 cells were observed under an inverted microscope. Cell proliferation was assessed using an MTT assay, cell cycles were analyzed using flow cytometry, and apoptosis was detected by Annexin V/PI staining. The expression of TOPO I and TOPO II*α* was analyzed by Western blot techniques, and their activity was measured using the TOPO I-mediated, supercoiled pBR322 DNA relaxation and TOPO II*α*-mediated Kinetoplast DNA (kDNA) decatenation assays, respectively. Supercoiled pBR322 and kDNA were also used to determine the direct effect of *β*-ELE on DNA breaks. *Results*. *β*-ELE significantly inhibited HepG-2 cell proliferation in a dose- and time-dependent manner. *β*-ELE also induced tumor cell arrest at S phase, induced cell apoptosis, and downregulated the protein expression of TOPO I and TOPO II*α* in a dose-dependent manner. *β*-ELE also inhibited TOPO I- and TOPO II*α*-mediated DNA relaxation but did not directly induce DNA breakage at any concentration. *Conclusion*. *β*-ELE could inhibit the proliferation of HepG-2 cells and interfere with the expression and activity of TOPO I and TOPO II*α*.

## 1. Introduction

Until now, no survival benefit has been demonstrated with any chemotherapy (systemic treatment) for patients with hepatocellular carcinoma (HCC) [1, 2]. Although sorafenib is the first drug to modestly improve the survival of patients with HCC, more appropriate use of this drug is recommended because of serious adverse events and deaths that have occurred in Japan [3]. An efficient drug against hepatocarcinoma for the therapy of HCC is anxiously anticipated.

**Figure 1:**
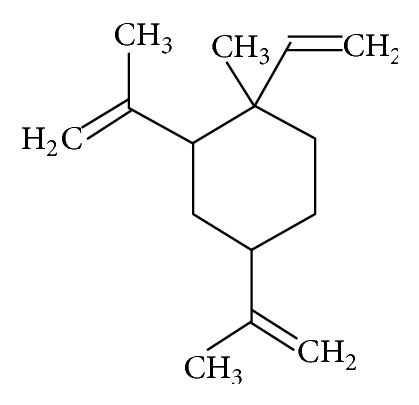
Structure of *β*-Elemene.

*β*-Elemene (beta-1-methyl-1-vinyl-2,4-di-isopropenyl-cyclohexane; *β*-ELE), a noncytotoxic antitumor element isolated from the traditional Chinese herbal medicine Rhizoma Zedoariae (Figure 1), has been approved by the State Food and Drug Administration of China for tumor therapy for decades. As a noncytotoxic agent with high antitumor efficacy and low cellular toxicity to normal tissues, *β*-ELE has been commonly used in Chinese clinical practice and has exhibited a broad spectrum of antitumor activity. Furthermore, *β*-ELE demonstrates efficacy in tumors that are unresponsive to chemotherapy, such as hepatocarcinoma, brain gliomas, lung cancer, ovarian carcinoma, and breast cancer [4–8]. No bone marrow suppression or drug resistance has been reported in clinical observations; on the contrary, the immunity and quality of life of patients improved during *β*-ELE therapy [9]. Clinical data also showed synergistic effects of *β*-ELE in combination with chemotherapy. *β*-ELE is now being evaluated in clinical trials in the United States [10]. Basic studies illustrated that *β*-ELE could pass through the blood-brain barrier [11] and increase tumor cell immunogenicity, at least in part, by inducing elevated expression of heat shock protein 70 on the tumor cell surface [9] and circumventing chemoresistance [12]. However, no data have shown the effect of *β*-ELE on topoisomerases. Topoisomerases, including TOPO I and TOPO II, are key enzymes in the regulation of nucleic acid topology configurations, and topoisomerases have become important targets for antitumor drugs [13]. Mammalian cells express two types of TOPO II enzymes, TOPO II*α* and TOPO II*β*, but only TOPO II*α* is essential for cellular viability due to its fundamental role in DNA metabolism and chromatin organization during interphase and mitosis. Moreover, TOPO II*α* is one of the most important drug targets in cancer chemotherapy [1, 2, 14].

This study observed the impact of *β*-ELE on human hepatocarcinoma HepG-2 cells, including its effect on cell proliferation, apoptosis, and the expression and activity of TOPO I and TOPO II*α*. We found that *β*-ELE could inhibit the proliferation of HepG-2 cells and induce tumor cell apoptosis. The downregulation of TOPO I and TOPO II*α* expression and activity might contribute to the inhibitory nature of *β*-ELE for HCC.

## 2. Materials and Methods

### 2.1. Drugs and Reagents

Injectable *β*-ELE was purchased from Dalian Holley King Kong Pharmaceutical Co., Ltd. (Dalian, China, lot number: 0508031); hydroxycamptothecin (HCPT) was purchased from Shenzhen Wandong Pharmaceutical Industry Co., Ltd. (Shenzhen, China); etoposide injection (VP-16) was purchased from Jiangsu Hengrui Medicine Co., Ltd. (Jiangsu, China); Iscove's Modified Dulbecco's Medium (IMDM), fetal bovine serum (FBS, Qualified), and 0.25% trypsin were purchased from Gibco (Life Technologies, USA); 3-(4,5-dimethylthiazol-2-yl)-2,5-diphenyltetrazolium bromide (MTT) was purchased from Biosharp (Hefei, China); an Annexin V (AV) kit was purchased from Molecular Probes (Life Technologies, USA); propidium iodide (PI) was purchased from Sigma Chemical Co. (St. Louis, USA); ethidium bromide was purchased from Amresco (OH, USA); a TRIzol kit was purchased from Invitrogen (Life Technologies, USA); chloroform, isopropyl alcohol, and ethanol were analytical grade reagents that were made in China; sodium lauryl sulfate, tetramethylethylenediamine, acrylamide, and methylenebisacrylamide were purchased from Sigma (St. Louis, USA); glycine was purchased from Shanghai Sangon Biotech Co., Ltd. (Shanghai, China); 1 M Tris (pH 6.8), 1.5 M Tris (pH 8.8), and Beyo-Enhanced Chemiluminescence (ECL) Plus were purchased from Beyotime Institute of Biotechnology (Shanghai, China); a bicinchoninic acid (BCA) protein assay kit was purchased from KeyGEN Biotech (Nanjing, China); rabbit anti-human TOPO I polyclonal antibody (sc-10783) and rabbit anti-human TOPO II*α* polyclonal antibody (sc-13058) were from Santa Cruz (Santa Cruz, CA, USA); and horseradish peroxidase-conjugated goat anti-rabbit IgG II was purchased from Hebei Bio-high Technology Deve Co., Ltd. (Hebei, China). PBR322 deoxyribonucleic acid (DNA) and DNA-TOPO I were purchased from Takara Biotechnology (Dalian) Co., Ltd. (Dalian, China); kDNA and DNA-TOPO II*α* were purchased from Vaxron Corporation (Rockaway, NJ, USA).

### 2.2. Cell Lines and Cultures

The human hepatocarcinoma cell line HepG-2 was provided by the Central Laboratory of the First Affiliated Hospital of Dalian Medical University, Dalian, China. The cells were incubated in low-carbohydrate IMEM medium supplemented with 10% FBS and cultivated in a humidified atmosphere with 5% CO_2_ at 37°C. The culture media were replaced on every 2nd day, and the cells were digested and passaged using 0.25% trypsin. When the growth of the HepG-2 cells was approximately 90% complete and a monolayer had formed, cells in the logarithmic growth phase were collected for the subsequent experiments.

### 2.3. Inverted Microscope for Cell Morphological Observation

HepG-2 cells in the logarithmic growth phase were treated with *β*-ELE at different concentrations (0, 20, 40, 60, 80, and 100 *μ*g/mL) and incubated for 72 h. Both the control (*β*-ELE at a concentration of 0 *μ*g/mL) and treated groups were observed under an inverted microscope (OLYMPUS, Japan) at 40x magnification and photographed. The experiment was repeated 3 times.

### 2.4. MTT Assay for *β*-ELE Cytotoxicity

Cell proliferation was evaluated using an MTT colorimetric assay. HepG-2 cells in logarithmic growth were plated at a density of 5 × 10^3^ cells/well in 96-well plates and incubated in a CO_2_ incubator at 37°C to allow the cells to adhere. One column contained only culture medium and served as a blank control, and another column contained cells without drug to serve as an untreated control for each plate. After 24 h of incubation in culture medium, different concentrations of *β*-ELE (20, 40, 60, 80, 100, 120, 140, 160, 180, and 200 *μ*g/mL) were added to the treated group, and the cells were incubated for an additional 24, 48, and 72 h. For each drug concentration, 8 repeat wells were used. Next, 20 *μ*L of MTT reagent solution (5 g/L) was added to each well, and the cells were incubated for 4 h at 37°C. After the medium and MTT were removed, 150 *μ*L of dimethyl sulfoxide (DMSO) was added to each well, and the plate was placed on a plate shaker for 6 min at room temperature to dissolve water-insoluble formazan. Finally, the optical density (OD) was monitored at 570 nm using a Bio-Rad Model 680 microplate reader (Shanghai Touching Technology Co., Ltd., China). The inhibition rate (IR) was calculated using the following formula: IR% = [1 − ODtreated/Dcontrol] × 100%, where ODtreated is the mean OD of the experimental sample, ODblank is the mean OD of the blank control group, and ODcontrol is the mean OD of the untreated control group. The IC_50_ (concentration of drug that inhibits cell growth by 50%) values were calculated using SPSS 11.5. All experiments were performed at least 3 times, and representative data are presented.

### 2.5. Flow Cytometry (FCM) Analysis for Cell Cycle Distribution and Apoptosis

Cells were seeded in 25 cm^2^ culture flasks at a density of 5 × 10^5^ cells/mL and incubated for 24 h. Then, the cells were treated with *β*-ELE at concentrations of 0, 20, 40, and 60 *μ*g/mL for 24 h and 48 h.

The attached cells were then harvested by trypsinization, washed twice with ice-cold phosphate-buffered saline (PBS), and then fixed in ice-cold 70% ethanol overnight at −20°C. The samples were washed twice with PBS and then resuspended to a concentration of 1 × 10^6^ cells/mL and incubated with 20 *μ*g/mL RNaseA (Boehringer Mannheim, Indianapolis, IN) and 10 *μ*g/mL propidium iodide (PI) for 30 min in the dark at room temperature to allow maximum labeling of DNA. Finally, the samples were analyzed for cell cycle distribution using a FACS flow cytometer (FCM, Beckman Coulter, USA), and the data were analyzed using the CellQuest Software (Becton Dickinson, San Jose, CA, USA). The experiment was repeated 3 times.

To assess apoptosis, cells were washed twice with ice-cold phosphate-buffered saline (PBS, pH 7.4), resuspended in a binding buffer (10 mM HEPES, pH 7.4, 140 mM NaCl, and 2.5 mM CaCl_2_), and incubated with 10 *μ*L/mL AV and 5 *μ*L/mL PI for 15 min at room temperature in the dark. AV and PI fluorescence was monitored by FCM, and the data were analyzed using the CellQuest Software. The experiment was repeated 3 times.

### 2.6. Western Blot Analysis for the Expression of TOPO I and TOPO II*α* Proteins

Cells in the exponential growth phase were seeded into four 25 cm^2^ culture flasks at a density of 5 × 10^5^ cells/mL and incubated for 24 h. Afterward, the cells were treated with *β*-ELE at different concentrations (0, 20, 40, and 60 *μ*g/mL) for 48 h, washed thrice with ice-cold PBS, and lysed with prepared lysis buffer for 30 min on ice. Debris was then removed by centrifugation at 12,000 rpm for 12 min. The protein concentrations of the supernatants were determined using the BCA protein assay kit. Equal amounts of protein were subjected to electrophoresis using sodium dodecyl sulfate-polyacrylamide gel electrophoresis (SDS-PAGE, 5% stacking gel + 8% separating gel) and then transferred to polyvinylidene difluoride (PVDF) membranes. The membranes were first incubated in blocking solution (5% skim milk) for 1 h and then incubated overnight with primary antibody (rabbit anti-human polyclonal antibody) at 4°C. Afterward, the membrane was washed 3 times with Tris-buffered saline with Tween-20 (TBST; 10 mM Tris-HCl, 0.15 M NaCl, 8 mM sodium azide, 0.05% Tween-20, and pH 8.0), incubated with horseradish peroxidase-conjugated secondary antibody (goat anti-rabbit IgG) for 1 h at room temperature, and then washed with TBST 3 more times. Finally, the protein bands were visualized on Kodak X-ray film using an ECL system. The intensity ratios of the bands compared with control bands were analyzed using ImageJ software. The experiment was repeated 3 times.

### 2.7. TOPO I-Mediated Supercoiled pBR322 DNA Relaxation Assay

DNA TOPO I activity was determined using the supercoiled pBR322 DNA relaxation assay. The experiments were performed by incubating DNA TOPO I with 0.5 *μ*g supercoiled pBR322 DNA in 2 *μ*L of relaxation buffer under increasing concentrations (10, 20, 40, 60, 80, and 100 *μ*g/mL) of *β*-ELE. In this experiment, HCPT, a known TOPO I inhibitor, was used as a positive control. Reactions were incubated at 37°C for 30 min and terminated by adding 1 *μ*L stop solution (1% SDS, 50% glycerol, and 0.05% bromophenol blue). Samples were subjected to electrophoresis in 1.5% agarose gels for 30 min, and DNA was stained with 0.5 *μ*g/mL EB and photographed under a UV transilluminator. The experiment was repeated 3 times.

### 2.8. The Direct Effect of *β*-ELE on pBR322 DNA

Reaction buffer (2 *μ*L) containing 0.5 *μ*g of pBR322 DNA was incubated with increasing concentrations (10, 20, 40, 60, 80, and 100 *μ*g/mL) of *β*-ELE. In this experiment, pBR322 DNA was used as a negative control. Reactions were incubated at 37°C for 30 min and terminated by adding 1 *μ*L of stop solution. Samples were subjected to electrophoresis in 1.5% agarose gels, and DNA was stained and photographed as above. The experiment was repeated 3 times.

### 2.9. TOPO II*α*-Mediated Kinetoplast DNA (kDNA) Decatenation Assay

TOPO II*α* catalytic activity was measured using the kDNA decatenation assay. The experiments were performed by incubating DNA TOPO II*α* with 0.4 *μ*g kDNA in 2 *μ*L of reaction buffer with increasing concentrations (10, 20, 40, 60, 80, and 100 *μ*g/mL) of *β*-ELE. In this experiment, etoposide, a known TOPO II*α* inhibitor, was used as a positive control. The reactions were incubated at 37°C for 30 min and terminated by adding 1 *μ*L of stop solution (1% SDS, 50% glycerol, and 0.05% bromophenol blue). Samples were subjected to electrophoresis in 1.5% agarose gels for 1 h, and DNA was stained with 0.5 *μ*g/mL ethidium bromide (EB) and photographed under a UV transilluminator. The experiment was repeated 3 times.

### 2.10. The Direct Effect of *β*-ELE on kDNA

Reaction buffer (2 *μ*L) containing 0.4 *μ*g kDNA was incubated with increasing concentrations (10, 20, 40, 60, 80, and 100 *μ*g/mL) of *β*-ELE. In this experiment, kDNA was used as a negative control. The reactions were incubated at 37°C for 30 min and terminated by adding 1 *μ*L of stop solution. Samples were subjected to electrophoresis in 1.5% agarose gels, and DNA was stained and photographed as above. The experiment was repeated 3 times.

### 2.11. Statistical Analysis

The SPSS 11.5 statistical software was used for data analysis, and normally distributed data are shown as the mean number ± standard deviation (*x* ± *s*). The group values were compared using one-way analysis of variance, and experimental and control groups were compared using pair-wise Dunnett's test. *P* < 0.05 was considered statistically significant.

## 3. Results

### 3.1. The Impact of *β*-ELE on Cell Morphology

**Figure 2:**
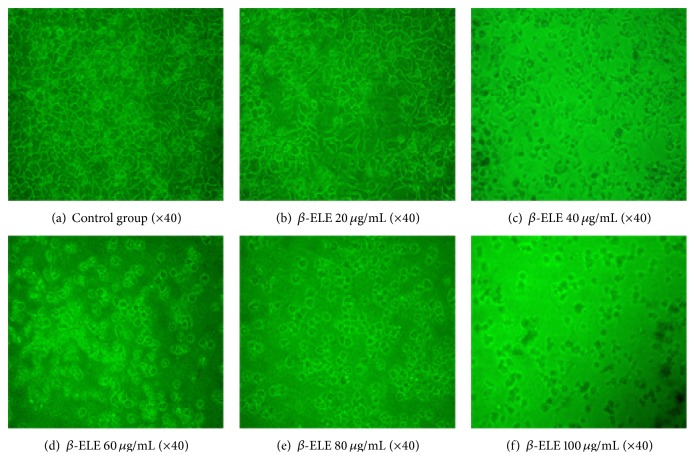
The role of *β*-ELE (*β*-Elemene) in cell morphological changes (×40). Figure 2 shows that *β*-ELE plays a role in the morphological changes of HepG-2 cells, as observed under an optical inverted microscope. (a) shows the control group, which is without *β*-ELE. (b)–(f) show cells exposed to different concentrations of *β*-ELE (20, 40, 60, 80, and 100 *μ*g/mL, resp.). Compared with the normal cells, the treated cells were markedly shrunken, the cell membrane was partially broken, and some cells showed necrolysis and partial karyopyknosis. These changes were more severe and more evident with increasing concentrations of *β*-ELE. The culture medium showed a large amount of cell debris and few intact cells when cells were treated with 100 *μ*g/mL.

Observation under an optical inverted microscope revealed that *β*-ELE induced morphological changes in HepG-2 cells, as shown in Figure 2. After 72 h of culture, cells of the control group were adherent, spindle-shaped, and tightly packed (Figure 2(a)). Compared with the control group, cells treated with different concentrations of *β*-ELE (20, 40, 60, 80, and 100 *μ*g/mL) were markedly shrunken, the cell membrane was partially broken, and some cells showed necrolysis and partial karyopyknosis. These changes were more severe or more evident with increasing concentrations of *β*-ELE (Figures 2(b)–2(f)). The culture medium showed a large amount of cell debris, and few cells were intact when treated with 100 *μ*g/mL.

### 3.2. Effects of *β*-ELE on the Proliferation of HepG-2 Cells

**Figure 3:**
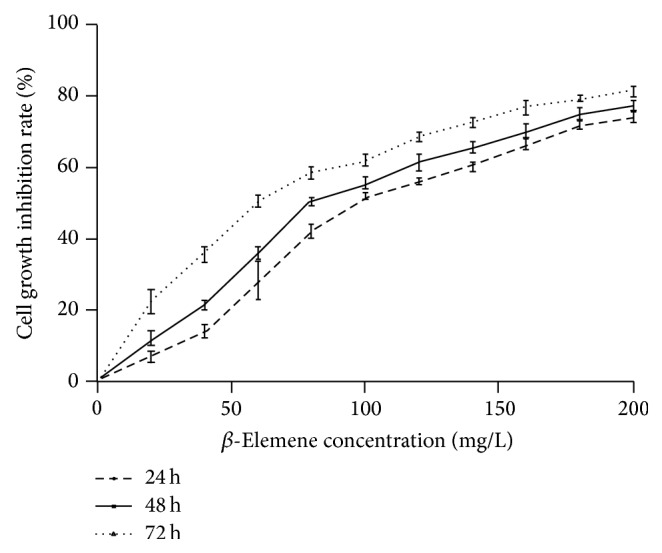
The effects of *β*-ELE (*β*-Elemene) on the proliferation of human hepatocarcinoma HepG-2 cells. Figure 3 shows the effect of different doses of *β*-ELE on cell proliferation at different time periods. *β*-ELE significantly decreased the growth of HepG-2 cells compared with the control group. The IC_50_s were 96.13, 80.84, and 60.95 *μ*g/mL for 24, 48, and 72 h, respectively, and the inhibitory effect was dose- and time-dependent.

As shown in Figure 3, the cell proliferation rate was affected by increasing *β*-ELE concentrations at different time intervals. *β*-ELE significantly decreased the growth of HepG-2 cells compared with the control group. The IC_50_s were 96.13, 80.84, and 60.95 *μ*g/mL for 24, 48, and 72 h, respectively, and the inhibitory effect was dose- and time-dependent. These data indicate that *β*-ELE has an inhibitory effect on the proliferation of HepG-2 cells.

### 3.3. *β*-ELE Induces Cell Cycle Arrest and Apoptosis in HepG-2 Cells

**Figure 4:**
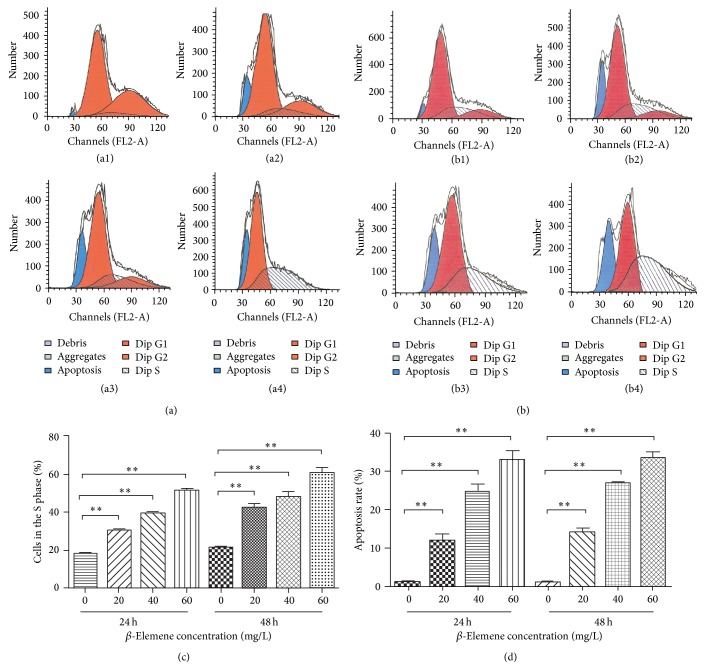
*β*-ELE induces cell cycle arrest and apoptosis in HepG-2 cells. (a) The effect of *β*-ELE (*β*-Elemene) on the cell cycle of HepG-2 cells after 24 h. (a1) Control group (without *β*-ELE). (a2) Experimental group (*β*-ELE 20 *μ*g/mL). (a3) Experimental group (*β*-ELE 40 *μ*g/mL). (a4) Experimental group (*β*-ELE 60 *μ*g/mL). After treatment with different concentrations of *β*-ELE (0, 20, 40, and 60 *μ*g/mL) for 24 h, a significant S phase arrest in HepG-2 cells was observed. The percentage of cells in S phase was 30.40 ± 1.19%, 39.38 ± 0.93%, and 51.43 ± 1.68% at 24 h when treated with *β*-ELE at 20, 40, and 60 *μ*g/mL, respectively. These values were apparently higher than that of the control group after 24 h (18.29 ± 0.94%, *P* < 0.05). (b) The effect of *β*-ELE (*β*-Elemene) on the cell cycle of HepG-2 cells after 48 h. (b1) Control group (without *β*-ELE). (b2) Experimental group (*β*-ELE 20 *μ*g/mL). (b3) Experimental group (*β*-ELE 40 *μ*g/mL). (b4) Experimental group (*β*-ELE 60 *μ*g/mL). After treatment with different concentrations of *β*-ELE (1, 20, 40, and 60 *μ*g/mL) for 48 h, a significant S phase arrest in HepG-2 cells was observed. The percentage of cells in S phase was 42.36 ± 3.40%, 47.86 ± 4.83%, and 60.95 ± 4.61% after 48 h when treated with *β*-ELE at 20 *μ*g/mL, 40 *μ*g/mL, and 60 *μ*g/mL, respectively. These values were apparently higher than that of the control group at 48 h (21.47 ± 0.59%, *P* < 0.05). (c) *β*-Elemene induces human hepatocarcinoma HepG-2 cell arrest at S phase. After treatment with different concentrations of *β*-ELE (0, 20, 40, and 60 *μ*g/mL) for 24 h and 48 h, a significant S phase arrest in HepG-2 cells was observed. The percentage of cells in S phase was 30.40 ± 1.19%, 39.38 ± 0.93%, and 51.43 ± 1.68% after 24 h and 42.36 ± 3.40%, 47.86 ± 4.83%, and 60.95 ± 4.61% after 48 h when treated with *β*-ELE at 20, 40, and 60 *μ*g/mL, respectively. These values were apparently higher than those of the control group at 24 h (18.29 ± 0.94%, *P* < 0.05) (Figure 4) and 48 h (21.47 ± 0.59%, *P* < 0.05) (Figure 5), which suggests that *β*-ELE treatment leads to an accumulation of HepG-2 cells in S phase (^*∗∗*^ *P* < 0.005). (d) The effect of *β*-ELE (*β*-Elemene) on the apoptosis of human hepatocarcinoma HepG-2 cells. After treatment with different concentrations of *β*-ELE (20, 40, and 60 *μ*g/mL) for 24 h and 48 h, the percentage of apoptosis cells was 11.94 ± 1.6%, 24.61 ± 2.07%, and 32.81 ± 2.58% after 24 h and 14.69 ± 1.77%, 27.14 ± 0.87%, and 34.38 ± 2.61% after 48 h. These values were significantly higher than those of the control group at 24 h (0.82 ± 0.27%, *P* < 0.05) and 48 h (1.07 ± 0.35%, *P* < 0.05) and showed dose and time dependence (^*∗∗*^ *P* < 0.005).

A significant S phase arrest in HepG-2 cells induced by *β*-ELE was observed (Figure 4). The percentage of cells in S phase was 30.40 ± 1.19%, 39.38 ± 0.93%, and 51.43 ± 1.68% after 24 h and 42.36 ± 3.40%, 47.86 ± 4.83%, and 60.95 ± 4.61% after 48 h, when treated with 20, 40, and 60 *μ*g/mL of *β*-ELE, respectively. All of these values were apparently higher than those of the control group after 24 h (18.29 ± 0.94%, *P* < 0.05) (Figure 4(a)) and 48 h (21.47 ± 0.59%, *P* < 0.05) (Figure 4(b)), which suggests that *β*-ELE treatment leads to an accumulation of HepG-2 cells in S phase (Figure 4(c)).

In addition, the percentage of apoptotic cells was 11.94 ± 1.6%, 24.61 ± 2.07%, and 32.81 ± 2.58% after 24 h and 14.69 ± 1.77%, 27.14 ± 0.87%, and 34.38 ± 2.61% after 48 h when cells were treated with 20, 40, and 60 *μ*g/mL *β*-ELE. These values were significantly higher than those of the control group after 24 h (0.82 ± 0.27%, *P* < 0.05) and 48 h (1.07 ± 0.35%, *P* < 0.05) and showed dose and time dependence (Figure 4(d)). These results suggest that *β*-ELE can effectively induce S phase arrest and apoptosis in HepG-2 cells.

### 3.4. Effects of *β*-ELE on TOPO I and TOPO II*α* Protein Expression in HepG-2 Cells

**Figure 5:**
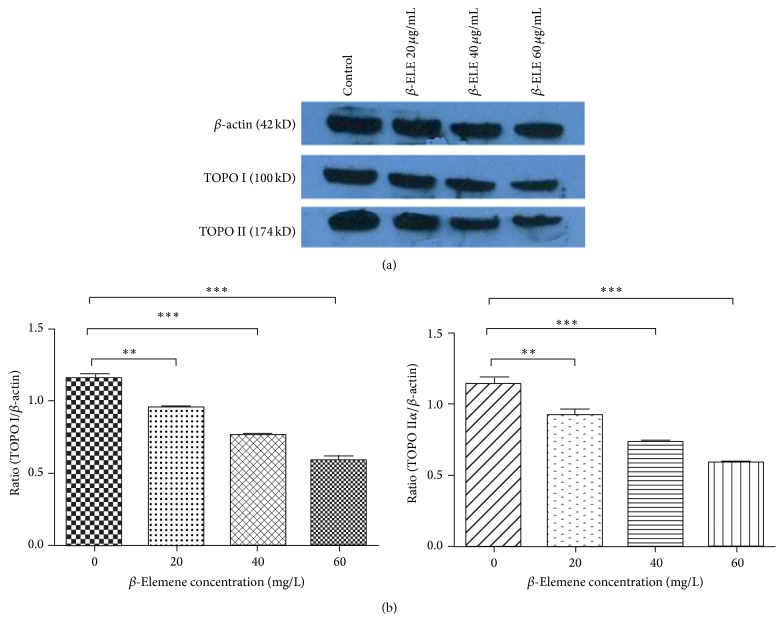
The effects of *β*-ELE (*β*-Elemene) on TOPO I and TOPO II*α* protein expression in HepG-2 cells. (a) Western blot analysis showed that both the ratios of TOPO I/*β*-actin (0.960 ± 0.036, 0.759 ± 0.034, and 0.591 ± 0.049) and TOPO II*α*/*β*-actin (0.937 ± 0.029, 0.752 ± 0.015, and 0.600 ± 0.017) were significantly decreased in a dose-dependent manner after *β*-ELE treatment (0, 20, 40, and 60 *μ*g/mL, *P* < 0.05). (b) The ratios of TOPO I/*β*-actin (0.960 ± 0.036, 0.759 ± 0.034, and 0.591 ± 0.049) were significantly lower than that of the control group (1.161 ± 0.043); and the ratios of TOPO II*α*/*β*-actin (0.937 ± 0.029, 0.752 ± 0.015, and 0.600 ± 0.017) were significantly lower than that of the control group (1.134 ± 0.045) (^*∗∗*^ *P* < 0.005, ^*∗∗∗*^ *P* < 0.001). These results suggest that the expression of the TOPO I and TOPO II*α* proteins significantly decreased in a dose-dependent manner after *β*-ELE treatment.

After treatment with different doses of *β*-ELE for 48 h, a dose-dependent decrease in the protein expression of TOPO I and TOPO II*α* in HepG-2 cells was observed, as shown in Figure 5. The ratio of TOPO I/*β*-actin (0.960 ± 0.036, 0.759 ± 0.034, and 0.591 ± 0.049, resp.) was significantly lower than that of the control group (1.161 ± 0.043) (*P* < 0.05), and the ratio of TOPO II*α*/*β*-actin (0.937 ± 0.029, 0.752 ± 0.015, and 0.600 ± 0.017, resp.) was significantly lower than that of the control group (1.134 ± 0.045) (*P* < 0.05). These results suggest that the expression of TOPO I and TOPO II*α* proteins significantly decreased in a dose-dependent manner after *β*-ELE treatment.

### 3.5. TOPO I Catalytic Activity Is Inhibited by *β*-ELE

**Figure 6:**
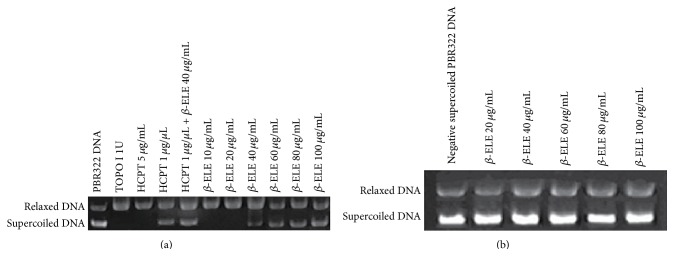
TOPO I catalytic activity is inhibited by *β*-ELE. (a) The effect of *β*-ELE (*β*-Elemene) on the relaxation of TOPO I-mediated, negative supercoiled pBR322 DNA. As shown in (a), lane 1 is negative supercoiled pBR322 DNA (supercoiled DNA, S); lane 2 is relaxed DNA that is the product of supercoiled DNA reacted with the enzyme (relaxed DNA, R); and lanes 3 and 4 are positive control groups. HCPT (a typical inhibitor of TOPO I) had no inhibitory effect on the DNA relaxation activity of TOPO I at the concentration of 5 *μ*g/mL, whereas it completely inhibited the relaxation of pBR322 DNA mediated by TOPO I at a concentration of 1 *μ*g/*μ*L. Lane 5 shows the effect of 1 *μ*g/*μ*L HCPT combined with 40 *μ*g/mL *β*-ELE on the relaxation of TOPO I-mediated negative supercoiled pBR322 DNA. As shown in (a), the combination inhibited the relaxation activity of TOPO I. Lanes 6 to 11 show the effects of different concentrations of *β*-ELE (40, 60, 80, and 100 *μ*g/mL) on the relaxation of negative supercoiled pBR322 DNA mediated by TOPO I. *β*-ELE has no inhibitory effect on the relaxation activity of TOPO I at concentrations of 10 and 20 *μ*g/mL; however, with increasing drug concentration, *β*-ELE showed an increasing inhibitory effect on the DNA relaxation activity of TOPO I at concentrations of 40, 60, 80, and 100 *μ*g/mL. The OD of MAX was 58 ± 3, 80 ± 6, 92 ± 10, and 134 ± 12. The statistical analysis showed significant differences between the 100 *μ*g/mL and 40 *μ*g/mL treatment groups and the 60 *μ*g/mL and 80 *μ*g/mL treatment groups (*P* < 0.05). (b) *β*-ELE (*β*-Elemene) has no direct effect on pBR322 DNA. As shown in (b), the average OD of the control group was 22860 ± 2412, and those of the *β*-ELE treatment groups were 24572 ± 518, 22318 ± 651, 22781 ± 837, 20781 ± 1180, and 24284 ± 749 for 20, 40, 60, 80, and 100 *μ*g/mL of *β*-ELE, respectively. There was no significant difference between the groups (*P* > 0.05).

DNA TOPO I regulates the number of topological links between two DNA strands (i.e., the change in the number of superhelical turns) by catalyzing transient single- or double-strand breaks, crossing the strands through one another, and then resealing the breaks, so that DNA converts to a relaxed state. Therefore, TOPO I can relax supercoiled DNA. The appearance of relaxed DNA bands and the disappearance of supercoiled forms are regarded as evidence of the catalytic activity of TOPO I. The effect of *β*-ELE on the catalytic activity of TOPO I was examined using the TOPO I-mediated supercoiled pBR322 DNA relaxation assay. As shown in Figure 6(a), lane 1 is negative supercoiled pBR322 DNA (supercoiled DNA, S); lane 2 is relaxed DNA that is the product of supercoiled DNA that has reacted with the enzyme (relaxed DNA, R); and lanes 3 and 4 are positive control groups. HCPT (a typical inhibitor of TOPO I) at 5 *μ*g/mL had no inhibitory effect on the DNA relaxation activity of TOPO I, whereas, at 1 *μ*g/*μ*L, it completely inhibited the relaxation of pBR322 DNA mediated by TOPO I. Lane 5 shows the effect of 1 *μ*g/*μ*L HCPT combined with 40 *μ*g/mL *β*-ELE on the relaxation of TOPO I-mediated, negative supercoiled pBR322 DNA. As shown in Figure 6(a), the combination inhibited the relaxation activity of TOPO I. Lanes 6 to 11 show the effects of different concentrations of *β*-ELE (40, 60, 80, and 100 *μ*g/mL) on the relaxation of negative supercoiled pBR322 DNA mediated by TOPO I. *β*-ELE had no inhibitory effect on the relaxation activity of TOPO I at concentrations of 10 and 20 *μ*g/mL; however, *β*-ELE showed an increasingly inhibitory effect on the DNA relaxation activity of TOPO I at increasing concentrations of 40, 60, 80, and 100 *μ*g/mL; the OD of MAX was 58 ± 3, 80 ± 6, 92 ± 10, and 134 ± 12, respectively. The statistical analysis showed significant differences between the 100 *μ*g/mL and 40 *μ*g/mL treatment groups and the 60 *μ*g/mL and 80 *μ*g/mL treatment groups (*P* < 0.05). These results demonstrate that *β*-ELE has an inhibitory effect on the catalytic activity of TOPO I, and the inhibition occurs in a dose-dependent manner.

To eliminate the effect of *β*-ELE on the relaxation of TOPO I-mediated, negative supercoiled pBR322 DNA, an experiment on the direct effect of *β*-ELE on negative supercoiled pBR322 DNA was conducted. As shown in Figure 6(b), the average OD of the control group was 22860 ± 2412, and those of the *β*-ELE treatment groups were 24572 ± 518, 22318 ± 651, 22781 ± 837, 20781 ± 1180, and 24284 ± 749 for 20, 40, 60, 80, and 100 *μ*g/mL of *β*-ELE, respectively. There was no significant difference between the groups (*P* > 0.05), which suggested that *β*-ELE has no direct effect on pBR322 DNA.

### 3.6. TOPO II*α* Catalytic Activity Inhibited by *β*-ELE

**Figure 7:**
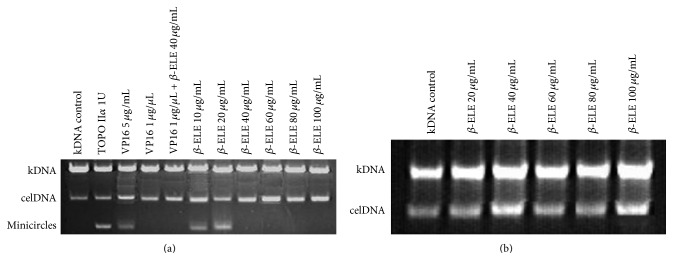
TOPO II*α* catalytic activity inhibited by *β*-ELE. (a) The effect of *β*-ELE (*β*-Elemene) on the decatenation of TOPO II*α*-mediated kDNA. As shown in (a), lane 1 is kDNA and served as a control group; lane 2 is the free DNA minicircles that were produced by the decatenation of TOPO II*α*, in this case, without *β*-ELE. Lanes 3 and 4 are the positive control groups. VP16 (a typical inhibitor of TOPO II*α*) at a concentration of 5 *μ*g/mL had no inhibitory effect on the DNA decatenation activity of TOPO II*α*, whereas DNA minicircles disappeared at a concentration of 1 *μ*g/*μ*L, which suggests that VP16 inhibits the catalytic activity of TOPO II*α*. Lane 5 shows the effect of 1 *μ*g/*μ*L VP16 with 40 *μ*g/mL *β*-ELE on the decatenation of TOPO II*α*-mediated kDNA. As shown in the figure, the activity of TOPO II*α* was inhibited. Lanes 6 to 11 show the effects of different concentrations (40, 60, 80, and 100 *μ*g/mL) of *β*-ELE on the decatenation of TOPO II*α*-mediated kDNA. (b) *β*-ELE (*β*-Elemene) has no direct effect on kDNA. As shown in (b), DNA minicircles, which form after DNA double-strand breakage, did not appear in the figure, suggesting that *β*-ELE has no direct effect on DNA.

KDNA is a strong network consisting of thousands of double-stranded, circular DNA. When one of the DNA minicircles is freed from the network structure, it must transfer a transiently double-stranded break to the main chain of the DNA. The decatenation assay is specific for measuring TOPO II*α* activity because it is based on the conversion of catenated DNA to its decatenated form, which requires the DNA double-strand break that is uniquely performed by TOPO II*α* [15]. The removal of these kDNA by the enzyme can be observed in agarose gels. In addition, with respect to TOPO II*α*, the decatenation of kDNA was induced by TOPO II*α* and inhibited by treatment with *β*-ELE, as shown in Figure 7(a). Lane 1 is the control kDNA; lane 2 is the free DNA minicircles that are produced by the decatenation of TOPO II*α*, in this case, without *β*-ELE; and lanes 3 and 4 are the positive control groups. VP16 (a typical inhibitor of TOPO II*α*) at a concentration of 5 *μ*g/mL had no inhibitory effect on the DNA decatenation activity of TOPO II*α*, whereas DNA minicircles disappeared at a concentration of 1 *μ*g/*μ*L, which suggested that VP16 inhibited the catalytic activity of TOPO II*α*. Lane 5 shows the effect of 1 *μ*g/*μ*L VP16 with 40 *μ*g/mL *β*-ELE on the decatenation of TOPO II*α*-mediated kDNA. As shown in Figure 7(a), the activity of TOPO II*α* was inhibited. Lanes 6 to 11 are the effects of different concentrations of *β*-ELE (40, 60, 80, and 100 *μ*g/mL) on the decatenation of TOPO II*α*-mediated kDNA.

To eliminate the effect of *β*-ELE on the decatenation of TOPO II*α*-mediated kDNA, the direct effect of *β*-ELE on kDNA was analyzed. As shown in Figure 7(b), DNA minicircles, which exist after DNA double-strand breaks, did not appear in the figure, suggesting that *β*-ELE has no direct effect on DNA.

## 4. Discussion

The mammalian topoisomerases are enzymes that play important roles in regulating the breakage and religation of DNA, providing swivel points for the transcription and replication of DNA, and facilitating the segregation of chromatids prior to mitosis [16]. TOPO I introduces single-strand breaks in DNA, whereas TOPO II*α* breaks both strands [17]. The independent contribution of each enzyme to these processes is sometimes difficult to assess [18]. The fact that topoisomerases are a key requirement in the mammalian cell division cycle makes them important targets for cancer chemotherapy [16]. Inhibitors of DNA topoisomerase (e.g., the TOPO I inhibitors topotecan, camptothecin, and irinotecan and TOPO II*α* inhibitors doxorubicin/adriamycin and etoposide) are among the most effective drugs that are available for tumor therapy in clinical practice [19].

In the present study, direct measurements of enzyme activity confirmed that TOPO I and TOPO II*α* were inhibited by *β*-ELE in a dose-dependent manner. Furthermore, *β*-ELE could downregulate the expression of TOPO I and TOPO II*α*. The majority of topoisomerase inhibitors show selectivity against TOPO I or TOPO II*α*, and only a small number of compounds can act against both enzymes and could have strong antitumor activity [19, 20]. Our research identified that *β*-ELE simultaneously targets TOPO I and TOPO II*α* and is the first drug available in clinical practice to hinder both enzymes. In this study, we found that *β*-ELE induced human hepatocarcinoma HepG-2 cell arrest at S phase (the phase of DNA synthesis), and flow cytometry analysis showed consistency in the percentages of apoptotic and S phase cells. The present results confirm the results of several published studies that showed that the sensitivity of cells to the cytotoxic effects of topoisomerase was the highest in late S and early G2 phase of the cell cycle [20] and prevented cells from progressing through S phase [21]. The cells progressing through S phase were selectively susceptible to apoptosis when treated with inhibitors of TOPO I and TOPO II*α* [19, 22]. The increased toxicity of a topoisomerase inhibitor toward S phase of the cell cycle is explained as follows: during DNA replication, the stabilization of cleavable complexes between DNA and topoisomerases by most TOPO I and TOPO II*α* inhibitors causes a collision between the progressing DNA replication fork and a stabilized complex and, in turn, conversion of the complex into secondary lesions that generate DNA lesions, thereby initiating cellular responses that include cell cycle arrest, DNA repair, and/or apoptosis [19, 23]. In the present study, *β*-ELE showed higher topoisomerase inhibition activity than the TOPO I inhibitor HCPT or TOPO II*α* inhibitor VP16. The research has suggested that *β*-ELE has synergistic effects and acts as a selective topoisomerase inhibitor, and several published studies have shown that compounds that simultaneously target both TOPO I and TOPO II*α* might have high antitumor activity. Some dual inhibitors have been advanced to clinical trials. The basis for the strong activity may depend on the complex pattern of activities that include the inhibition and poisoning of the two enzymes or some novel mechanism that is suggested in preclinical tests [16].

## 5. Conclusion

In the present study, we show that *β*-ELE inhibits the proliferation of HepG-2 cells and induces tumor cell arrest at S phase. The downregulation of the expression and activity of TOPO I and TOPO II*α* might contribute to the inhibition of cell proliferation by *β*-ELE in HCC. Further studies are needed to understand the factors that result in the inhibition of the two enzymes (TOPO I and TOPO II*α*) by *β*-ELE and the mechanism by which *β*-ELE blocks the cell cycle.
